# Survival prediction with radiomics for patients with IDH mutated lower-grade glioma

**DOI:** 10.1007/s11060-025-05006-z

**Published:** 2025-03-18

**Authors:** Alice Neimantaite, Louise Carstam, Tomás Gómez Vecchio, Ida Häggström, Tora Dunås, Francesco Latini, Maria Zetterling, Malin Blomstrand, Jiri Bartek, Margret Jensdottir, Erik Thurin, Anja Smits, Asgeir S. Jakola

**Affiliations:** 1 Department of Clinical Neuroscience, Institute of Neuroscience and Physiology, Sahlgrenska Academy, University of Gothenburg, Gothenburg, Sweden; 2 Department of Neurosurgery, Sahlgrenska University Hospital, Gothenburg, Sweden; 3 Institute of Health and Care Sciences, Sahlgrenska Academy, University of Gothenburg, Gothenburg, Sweden; 4 Department of Electrical Engineering, Chalmers University of Technology, Gothenburg, Sweden; 5 Department of Medical Radiation Sciences, University of Gothenburg, Gothenburg, Sweden; 6 Department of Medical Sciences, Section of Neurosurgery, Uppsala University Hospital, Uppsala, Sweden; 7 Department of Oncology, Institute of Clinical Sciences, Sahlgrenska Academy, University of Gothenburg, Gothenburg, Sweden; 8 Department of Oncology, Sahlgrenska University Hospital, Gothenburg, Sweden; 9 Department of Clinical Neuroscience, Karolinska Institute, Stockholm, Sweden; 10 Department of Neurosurgery, Karolinska University Hospital, Stockholm, Sweden; 11 Department of Neurosurgery, Rigshospitalet, Copenhagen, Denmark; 12 Department of Radiology, Sahlgrenska University Hospital, Gothenburg, Sweden

**Keywords:** Glioma, Survival analysis, Magnetic resonance imaging, Radiomics

## Abstract

**Purpose:**

Adult patients with diffuse lower-grade gliomas (dLGG) show heterogeneous survival outcomes, complicating postoperative treatment planning. Treating all patients early increases the risk of long-term side effects, while delayed treatment may lead to impaired survival. Refinement of prognostic models could optimize timing of treatment. Conventional radiological features are prognostic in dLGG, but MRI could carry more prognostic information. This study aimed to investigate MRI-based radiomics survival models and compare them with clinical models.

**Methods:**

Two clinical survival models were created: a preoperative model (tumor volume) and a full clinical model (tumor volume, extent of resection, tumor subtype). Radiomics features were extracted from preoperative MRI. The dataset was divided into training set and unseen test set (70:30). Model performance was evaluated on test set with Uno’s concordance index (c-index). Risk groups were created by the best performing model’s predictions.

**Results:**

207 patients with mutated IDH (mIDH) dLGG were included. The preoperative clinical, full clinical and radiomics models showed c-indexes of 0.70, 0.71 and 0.75 respectively on test set for overall survival. The radiomics model included four features of tumor diameter and tumor heterogeneity. The combined full clinical and radiomics model showed best performance with c-index = 0.79. The survival difference between high- and low-risk patients according to the combined model was both statistically significant and clinically relevant.

**Conclusion:**

Radiomics can capture quantitative prognostic information in patients with dLGG. Combined models show promise of synergetic effects and should be studied further in astrocytoma and oligodendroglioma patients separately for optimal modelling of individual risks.

**Supplementary Information:**

The online version contains supplementary material available at 10.1007/s11060-025-05006-z.

## Introduction

Adult-type diffuse lower-grade gliomas (dLGG) refer to IDH mutated (mIDH) astrocytoma and oligodendroglioma WHO grade 2 and 3 [1–3]. Treatment of dLGG is multimodal and includes surgery, radiotherapy, chemotherapy, and now also possibly mIDH inhibitors [4, 5].

The postoperative treatment planning in patients with dLGG is connected to prognostic factors. Such risk factors are typically a combination of patient, radiological, and molecular information (astrocytoma or oligodendroglioma) [5]. As a significant proportion of patients with dLGG live more than 15 years, early oncological treatment has the potential to cause long-term harm [6–8]. On the other hand, withholding early oncological treatment may miss a window of opportunity and be prognostically disadvantageous. As such, optimal timing of postoperative treatment for the individual can balance the risk of adverse events while aiming for long-term survival.

Different prognostic models have been frequently used over the years [9–13]. Following the Radiation Therapy Oncology Group (RTOG) 9802 trial [14], age and residual tumor after resection have been used in oncological treatment selection. However, the risk factor of higher age is confounded by IDH wild type gliomas in the old classification scheme. Still, in some centres, this has led to a large proportion of patients with mIDH dLGG receiving immediate postoperative treatment [15]. A recent large study in the molecular era confirmed molecular subgroup and MRI-defined tumor volume (pre- and postoperative) as important prognostic factors [16]. However, MRI carries more information that potentially can refine the prognostication in patients with dLGG [17].

With radiomics, the extraction of mathematical features from MRI can be used for prognostication purposes [18, 19]. Radiomics features go beyond classic image interpretation and may capture properties not directly seen by the eye. Another benefit is that MRI covers the entire tumor as well as the adjacent infiltrated brain [20–22], not only a small biopsy.

The aim of this study was to compare radiomics survival models to relevant clinical survival models. Importantly, our focus was to find out if the radiomics model offers prognostic information *in addition* to the clinical information, to better understand the net gain of more complex models.

## Materials and methods

### Patient population

Patients with glioma of WHO grade 2 and 3 with mIDH and known 1p19q status were included from three Swedish University Hospitals with population-based uptake areas. The patients had undergone primary surgery in the period from 2007 to 2020. Clinical data was extracted from electronical health records. Patients were only included if the following preoperative MRI sequences were available: T1 with contrast enhanced (T1c) and fluid attenuated inversion recovery (FLAIR).

The patients were divided into training and test sets with ratio 70:30 using the Python programming language version 3.8.3 (Python Software Foundation). For details, see the Supplementary material Section S.1.1.

### Clinical survival models

The definition of dLGG and their subtypes has changed over the years [1, 23, 24]. Thus, clinical variables associated with survival are somewhat heterogeneous in different patient cohorts [3, 6, 9–13, 16]. We decided to build prognostic models based on recent evidence, reflecting the situation today [16]. Two parallel survival models were created: a preoperative clinical model (preoperative tumor volume) and a full clinical model (preoperative tumor volume, extent of resection and tumor subtype) containing variables available in the early postoperative phase, prior to decision of oncological treatment strategy.

#### Image annotations

The tumor volume was quantified from tumor segmentation. The process of tumor segmentation was done semi-automatically using 3DSlicer [25], as described in previous work [26].

The extent of resection was defined as biopsy (no tumor reduction), partial resection, or complete resection (no FLAIR residue). Postoperative tumor volume was not available for all patients. For patients missing postoperative MRI, the extent of resection variable was decided from the surgical notes and later MRI. A sensitivity analysis on the full clinical model performance was made by replacing the extent of resection variable in the model with the postoperative tumor volume.

### Radiomics survival models

#### Image preprocessing

The preoperative MRI sequences and tumor segmentations were linearly registered to the MNI-space as previously described [27]. Registered images were visually controlled and re-registration by adjustment of registration parameters, or manual registration was applied when needed.

Four radiomics survival models were built by feature extraction from one of four chosen tumor-related volumetric zones: (1) tumor zone (segmentation), (2) the peritumoral zone − 5 to 5 mm around the tumor segmentation edge, (3) the peritumoral zone 0 to 10 mm outside the segmented tumor, (4) the peritumoral zone 10 to 20 mm outside the segmented tumor [20, 22]. Segmentations of the peritumoral zones were automatically extracted, see specifications and visualization of the zones in the Supplementary material Section S.1.2.

#### Image feature extraction, selection and learning

Radiomics features were extracted using pyradiomics [28]. The included features were shape features, first-order and second-order features (see the Supplementary material Section S.1.3 for all screened features). The features were calculated on images with no applied filters. Every feature was normalized [29] using z-score normalization, separately for training and test sets, and separately for every tumor and peritumoral zone and separately for both MRI sequences. The feature selection on the training set was applied by firstly removing features with variance < 0.01. Secondly, for each pair of features with a Spearman’s correlation *≥* 0.95, the feature with the highest mean correlation with all other features was removed. Thirdly, LASSO-Cox regression was applied for the last feature selection, learning and the creation of radiomics survival models. Specifications are supplied in the Supplementary material Section S.1.4.

### Survival model interpretability

The features from the best performing radiomics model were interpreted using the explanations in pyradiomics. Further, chosen survival models were explained using SHapley Additive exPlanations (SHAP) [30], which was also clinically interpreted. See specifications in the Supplementary material Section S.1.5.

### Statistics

IBM SPSS Statistics versions 29 or newer (IBM Corp., Armonk, NY, USA) were used for data evaluation and statistical testing. Data normality was evaluated visually, and statistically using the Kolmogorov-Smirnov normality test. Group comparisons were done using Mann-Whitney U-test for continuous data and Fisher’s exact test for categorical data. Clinical and combination survival models were created by Cox regression using the Python lifelines library [31], more details are available in the Supplementary material Section S.1.6.

#### Prognostication performance evaluation

Survival model performance was primarily evaluated using Uno’s concordance index (c-index) [32] in Python. The c-index ranges between zero and one, one meaning a perfect patient survival ranking ability. Uno’s c-index was also evaluated for patients with observed time up to 5 years (tau = 5), i.e. how well the model estimates in poor prognosis. A c-index value distribution was built by bootstrapping described in the Supplementary material Section S.1.7. The chosen survival models were statistically compared by applying Wilcoxon signed rank test on the models’ c-index distributions, for each c-index variant separately. To facilitate comparison with other studies, the model performance was also evaluated using the more traditionally applied Harrell’s c-index [33]. However, Harrell’s c-index is not ideal for data with high proportion of censoring. Uno’s c-index is designed to adjust for censoring, which was more appropriate for evaluating the model performance on our data.

#### High- and low-risk groups

Patients were divided into high- and low-risk groups using the calculated risk scores by the best performing survival model. Risk threshold to define the risk groups was calculated on the entire cohort by maximizing the separation between Kaplan-Meier survival curves using log-rank test statistic. A minimal risk group size was set to 33% of the cohort. Specifications are supplied in the Supplementary material Section S.1.8. The separation between the Kaplan-Meier survival curves of the resulting high- and low-risk groups was decided by the log-rank test.

## Results

### Patient characteristics

In total 207 patients with mIDH gliomas were included in the study. 143 patients from Sahlgrenska University Hospital, 39 patients from Uppsala University Hospital and 25 patients from Karolinska University Hospital. The cohort characteristics are supplied in the Supplementary Table S1. No significant differences were found between the training and test set characteristics, as reported in Table 1.

**Table 1:** Training and test demographics, patient characteristics, tumor characteristics, treatment and survival

Variable	Training (*n* = 144)	Test (*n* = 63)	*p*-value^a^
Age at surgery, median (Q1, Q3)	40.5 (33.0, 50.0)	38.0 (32.0, 50.0)	0.37
Age > 40, n (%)	72 (50.0)	27 (42.9)	0.37
KPS^b^ < 80 at admission, n (%)	24 (16.7)	12 (19.0)	0.69
Tumor volume, ml, median (Q1, Q3)	53.8 (27.5, 116.8)	54.8 (22.8, 81.9)	0.31
Surgery			
Biopsy, n (%)	11 (7.6)	7 (11.1)	0.43
Partial resection, n (%)	103 (71.5)	44 (69.8)	0.87
Complete resection, n (%)	30 (20.8)	12 (19.0)	0.85
WHO classification			
Grade 2, n (%)	99 (68.8)	44 (69.8)	1.00
Grade 3, n (%)	45 (31.3)	19 (30.2)	1.00
Astrocytoma, n (%)	72 (50.0)	35 (55.6)	0.55
Oligodendroglioma, n (%)	72 (50.0)	28 (44.4)	0.55
Oncological treatment			
Radiotherapy (within 6 months), n (%)	82 (56.9)	32 (50.8)	0.62
Chemotherapy (within 6 months), n (%)	72 (50.0)	26 (41.3)	0.29
Survival			
Censored, n (%)	109 (75.7)	47 (74.6)	0.32

^a^ Mann-Whitney U test, Fisher’s exact test, ^b^ Karnofsky performance status scale

### Survival model performance

Survival model results are presented in Table 2. Results using the more traditional c-index (Harrell’s) can be found in the Supplementary material Section S.2.2.

**Table 2:** Results for each survival model on the test set (*n* = 63)

Survival model	Selected features *N*	Test Uno’s c-index median (IQR)	Test Uno’s at t *≤* 5y median (IQR)
**Preoperative Clinical**	1	0.700 (0.120)	0.697 (0.117)
**Full Clinical**	3	0.714 (0.124)	0.738 (0.132)
**Radiomics** Tumor	4	0.754 (0.123)	0.776 (0.092)
**Radiomics** PTZ *±* 5 mm	4	0.663 (0.132)	0.655 (0.130)
**Radiomics** PTZ 0–10 mm	1	0.725 (0.145)	0.755 (0.103)
**Radiomics** PTZ 10–20 mm	30	0.663 (0.123)	0.707 (0.127)
**Combined**: Preop Clinical + Radiomics	1 + 4	0.769 (0.117)	0.799 (0.088)
**Combined**: Full Clinical + Radiomics	3 + 4	0.793 (0.116)	0.813 (0.090)

The radiomics models with best c-index values were the radiomics model based on the tumor zone, followed by the radiomics model based on the peritumoral zone 0–10 mm. The best performing radiomics tumor zone model will be referred to as the radiomics model in the text below and this was the model which was combined with the clinical models.

#### Statistical comparison: radiomics compared to clinical models

The models’ c-index distributions were compared pairwise using Wilcoxon signed rank test, separately for Uno’s c-index and Uno’s c-index at 5 years. The radiomics model showed statistically significantly better performance than both clinical models in overall survival prediction (Uno’s) and in short-term prediction (Uno’s at 5y). All comparisons *p* < 0.001.

#### Statistical comparison: combined models compared to radiomics and clinical models

Addition of radiomics features to each of the clinical models significantly improved the prediction performance. The combined models showed significantly better performance than radiomics alone as well. The combined full clinical and radiomics model showed significantly better performance than the combined preoperative clinical and radiomics model. Uno’s: *p* < 0.001, Uno’s at 5y: *p* < 0.001 for all comparisons.

#### Sensitivity analysis: postoperative tumor volume

The full clinical model was re-trained with extent of resection replaced with postoperative tumor volume where this was available (training *n* = 129, test *n* = 57). This model showed the performance of 0.631 for overall survival (Uno’s) and 0.745 for short-term survival (Uno’s at 5y) on test set. More details are presented in the Supplementary material Section S.2.3.

### Survival model interpretability

#### Interpretation of the radiomics features

**Fig. 1:**
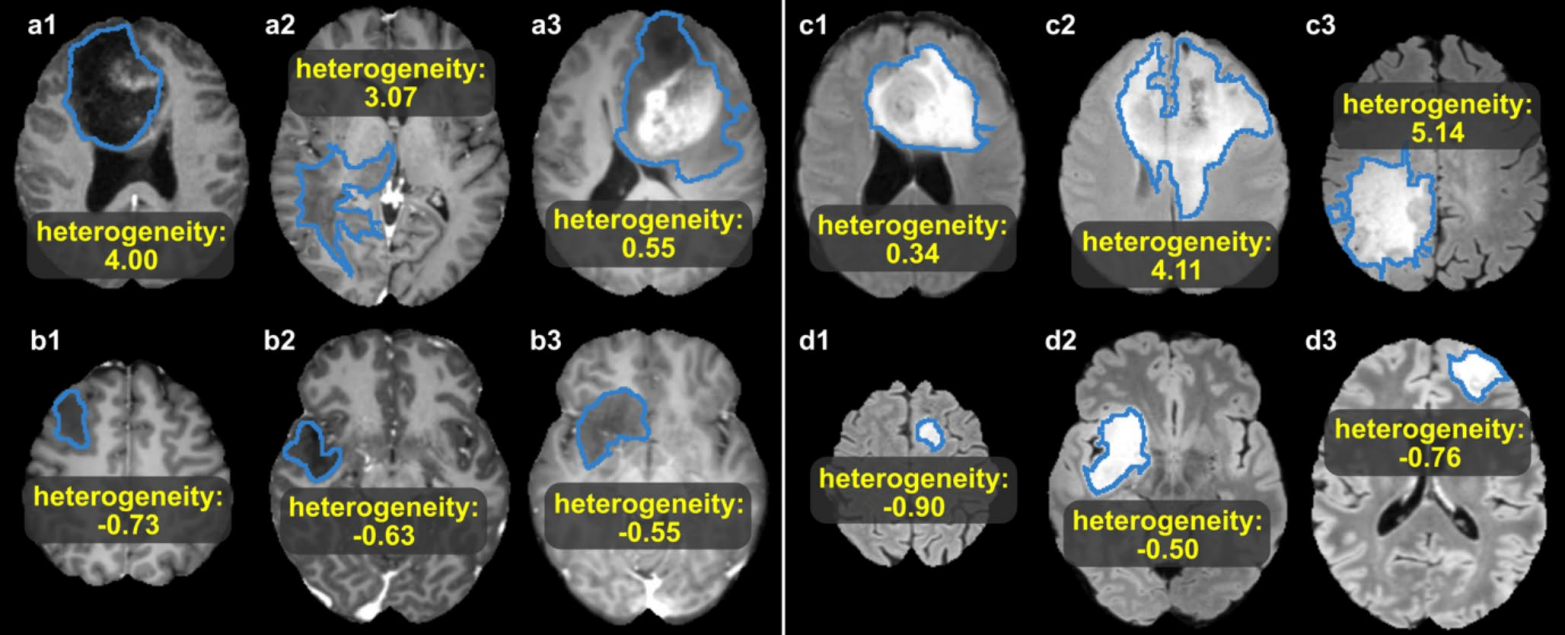
Visualization of 12 case examples of the radiomics tumor heterogeneity feature. (**a1-3**) visualizes patients with high values of the feature on T1c, (**b1-3**) patients with low values on T1c. (**c1-3**) visualizes patients with high values of the feature on FLAIR, (**d1-3**) patients with low values on FLAIR. Note that segmentations are done in FLAIR/T2-weighted images and may appear larger than the volume depicted in T1c images

The radiomics model resulted in four optimal features for survival prediction (the technical names are provided in the Supplementary material Section S.2.4). The two tumor diameter features were the largest 2D axial tumor diameter and the largest 3D tumor diameter (Feret diameter). The other two features are the same feature on T1c respectively FLAIR, which represents the amount of heterogeneity in the tumor based on the lengths of consecutive grey level pixel values in the image. Case examples of the heterogeneity feature are visualized in Fig. 1. For better readability in text, the four optimal radiomics features will be referred to as radiomics features of tumor diameter and tumor heterogeneity. The additional value of the diameter and heterogeneity features separately in the combined full clinical and radiomics model is provided in the Supplementary material Section S.2.5.

#### Interpretation of the models using SHAP

**Fig. 2:**
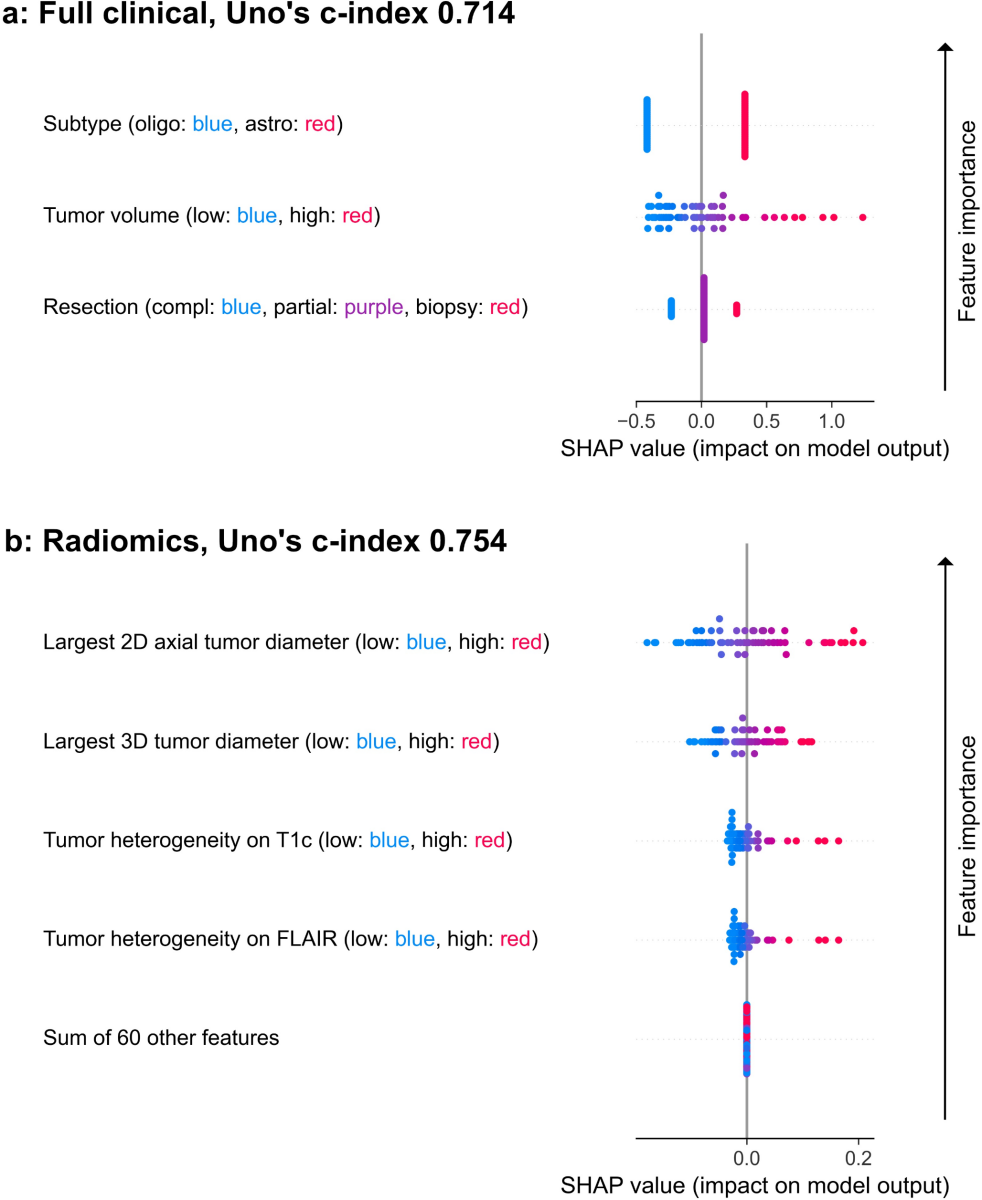
Survival model interpretability. Feature importance and distribution on the test set by the (**a**) full clinical survival model, and (**b**) radiomics survival model. Positive SHAP values indicate increased risk whereas negative values indicate reduced risk

SHAP results of feature contribution within the full clinical and the radiomics models are shown in Fig. 2. In the full clinical model, tumor subtype was the most impactful feature for risk prediction. Largest tumor volume values increased the patients’ risk more than the lowest values decreased the risk in the full clinical model. A similar trend was seen in the radiomics model regarding the tumor heterogeneity variables on T1c and FLAIR. The most impactful feature in the radiomics model was the largest 2D axial tumor diameter.

### Survival risk groups

**Fig. 3:**
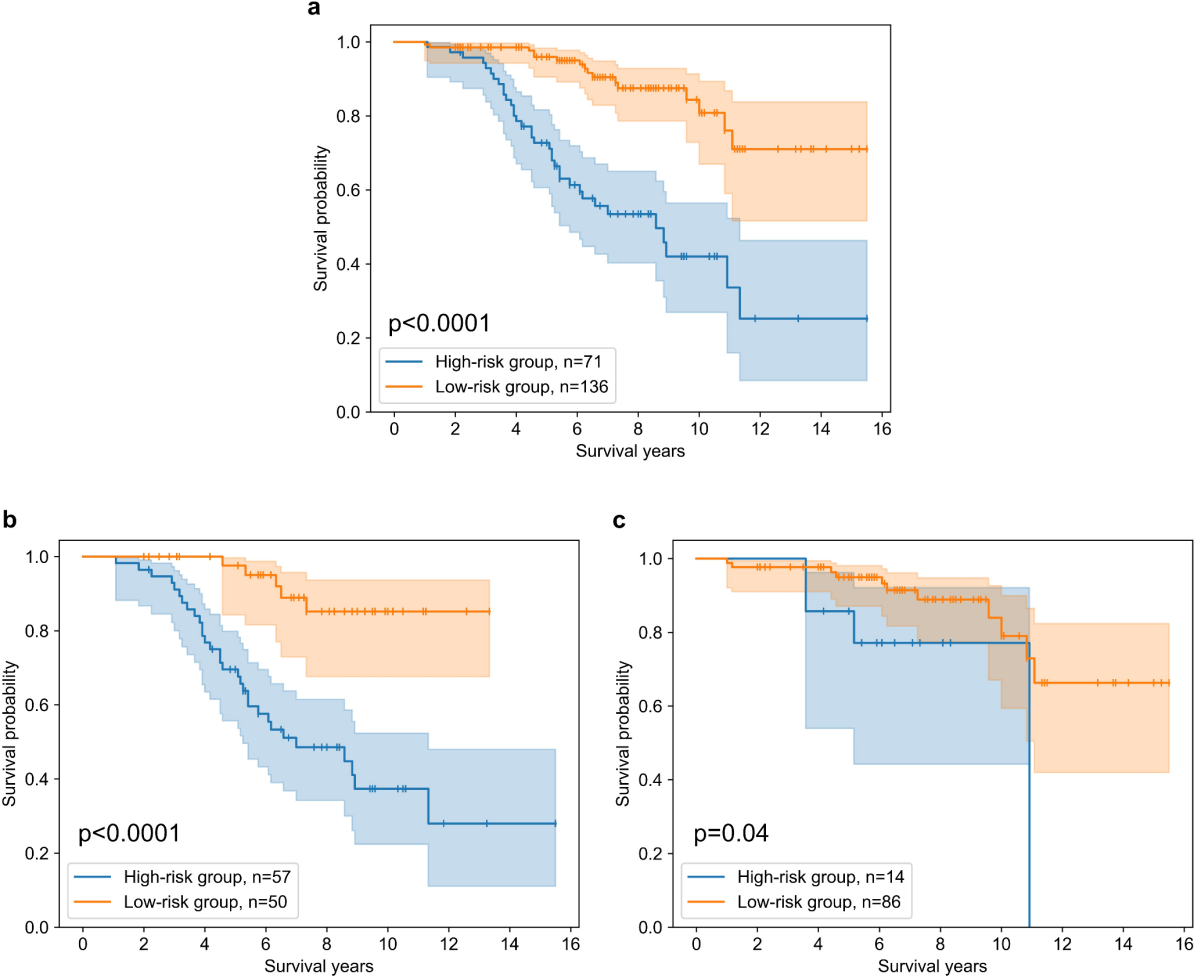
Kaplan-Meier survival curves of high- and low-risk patient groups within (**a**) entire patient cohort, (**b**) patients with astrocytoma, and (**c**) patients with oligodendroglioma

High- and low-risk patient groups were created using the combined full clinical and radiomics survival model’s risk scores. Kaplan-Meier plots for the entire cohort and within tumor subtypes are visualized in Fig. 3. Characteristics of the high- and low-risk groups of the cohort are supplied in the Supplementary Table S.5.

## Discussion

In this study, radiomics modelling identified preoperative MRI-based prognostic features with superior performance compared to clinical models. The radiomics features carrying prognostic information were related to tumor diameter and heterogeneity, and we present their relative importance compared to the traditional variables for patients with dLGG. Combining the early postoperative clinical features with radiomics features further improved the prognostic performance of the model. The combined model successfully stratified patients into high- and low-risk groups.

In previous studies, designed by the older WHO classification of dLGG and including a mixture of mIDH and IDH wild type gliomas, radiomics has performed well in survival prediction [34–36]. This was also seen in our study on mIDH dLGG. The features found by the radiomics model relate to recent findings in research and point to future directions to build an optimal model for prognostication in dLGG. The feature connected to tumor heterogeneity in our radiomics model is interesting. Histological tumor heterogeneity is a well-known concept in dLGG, imposing a limitation on biological analyses of tumor samples not taking the entire tumor volume into account. The MRI-based heterogeneity feature is therefore an inherent strength of radiomics. A recent study [37] investigated inter- and intra-observer variability in the subjective assessment of tumor heterogeneity on T2-weigthed MRI for tumor classification and found varying (moderate to very good) agreement. Quantitative heterogeneity measure as used in our study is potentially a superior method, as it is reproducible and unbiased. Our radiomics model selected heterogeneity on both FLAIR and T1c for the prognostic model. Qualitatively assessed contrast enhancement on T1c has been shown as a prognostic factor for dLGG in different tumor classification eras [38–40], and most recently it was found as prognostic for astrocytomas in particular [40]. Contrast enhancement might be reflected in the heterogeneity feature on T1c, although no clear indication for this is seen in Fig. 1.

The two tumor diameter features, largest 2D axial and largest 3D tumor diameters, were the strongest prognostic factors in the radiomics model. Although this may overlap considerably with volume in the clinical model, the combined model did show improved results. We hypothesize that this improvement could be due to importance of tumor shape and growth. Tumor shape has been found to offer prognostically relevant information in both glioblastomas and meningiomas [41, 42]. In both situations, a non-spherical (irregular) shape, calculated by the tumor surface area and volume, has shown to be a factor of unfavourable prognosis. In our study, the sphericity feature was not chosen by the radiomics selection process for dLGG prognostication. The relation between the 2D and 3D diameters and volume could reflect a branching shape, which might be an indirect measure of dLGG infiltration along the white matter tracts [21, 43]. In future studies on dLGG, we encourage to consider the prognostic impact of tumor shape and growth, rather than tumor volume alone.

Our results confirm that MRI features with clinically relevant information, beyond traditional image interpretation, can be extracted quantitatively and already at the preoperative phase for patients with dLGG. The combined model was able to identify high- and low-risk patients, which is of important clinical relevance. However, how to optimally combine the clinical and radiomics features should be investigated further in future studies. For potential clinical implementation, the radiomics feature extraction is judged as a relatively fast process once the software is set up, provided that segmentation of the tumor is available. The latter will likely become increasingly available, facilitated by automated segmentations [44].

The major strengths of our study are that the survival models have been based on a pure mIDH dLGG cohort, and that the results are presented in an unseen test set. Furthermore, the radiomics model has been compared to clinical models including factors accounted for in daily practice. Including both WHO grade 2 and 3 gliomas could potentially impact results. Although the literature is mixed concerning the impact of WHO grade 3 versus grade 2 in mIDH gliomas [3, 45–48], we and others have demonstrated that survival is strikingly similar [3, 45, 46], but treatment intensity may differ [3, 47]. Thus, we considered it more important to increase the sample size than to present WHO grades separately. Of note, the high- and low-risk groups in this study had similar proportions of WHO grade 3, indicating minimal impact. Another limitation is that the power in studying mortality in patients with dLGG was modest given the relatively long survival times in this group. For some of the longest living patients, postoperative tumor volume was not available, and the estimated extent of resection might have affected the clinical model performance. Furthermore, the highest event rate is in astrocytomas, and therefore the survival models may be more tuned to fit this subgroup. This is in line with most oligodendrogliomas being assigned to the low-risk group, and the stratification into high- and low-risk is therefore at present more useful in patients with astrocytomas. We used Uno’s c-index to counteract censoring in the model performance evaluation, yet the evaluation could be further improved by testing the model performance on an external and larger cohort. For future studies, we also recommend validating the prognostic variables within dLGG subtypes and using data with longer follow-up times, especially for patients with oligodendroglioma. Additionally, we encourage to explore radiomics on other MRI sequences and postoperative images in future studies for potential model refinement.

## Conclusions

Radiomics could identify MRI-based prognostic factors, which improved the prognostication performance. Beyond shape-related variables, heterogeneity of the tumor holds complementary prognostic information to the traditional variables in mIDH dLGG. Further research is needed to optimize prognostic models in dLGG to facilitate treatment planning in patients with dLGG.

## Electronic supplementary material

Below is the link to the electronic supplementary material.

Supplementary Material 1

## Data Availability

Data is available to be shared upon reasonable request.
