# Supplementary material for: Survival prediction with radiomics for patients with IDH mutated lower-grade glioma
Source: J Neurooncol. 2025 Mar 18;173(3):505–14. doi: 10.1007/s11060-025-05006-z (PMC12170745; doi:10.1007/s11060-025-05006-z)
Supplement: Supplementary file 1 — Supplementary Material 1 [file 11060_2025_5006_MOESM1_ESM.docx]

**Supplementary material**

**Journal: Journal of Neuro-Oncology**

**Article title:** Survival prediction with radiomics for patients with IDH mutated lower-grade glioma

**Authors:** Alice Neimantaite, Louise Carstam, Tomás Gómez Vecchio, Ida Häggström, Tora Dunås, Francesco Latini, Maria Zetterling, Malin Blomstrand, Jiri Bartek, Margret Jensdottir, Erik Thurin, Anja Smits, Asgeir S. Jakola

**Corresponding author:** [alice.neimantaite@neuro.gu.se](mailto:alice.neimantaite@neuro.gu.se)

Department of Clinical Neuroscience, Institute of Neuroscience and Physiology, Sahlgrenska Academy, University of Gothenburg, Gothenburg, Sweden

**S. 1. Methods**

**Python implementation – general**

Commonly used Python packages in the implementation were: pandas [1], matplotlib [2], numpy [3], scipy [4], and sksurv [5].

**S. 1.1. Methods: Patient division**

The patient cohort was split in a random manner, keeping the balance of tumor grades and censored patients within the training and test sets the same as within the entire cohort.

The function *train_test_split*(stratify=WHO_grade + Censored) from the sklearn.model_selection package [5] was applied.

**S. 1.2. Methods: Extraction of PTZ**

The peritumoral zones were created by dilation and erosion of the original segmentation using the functions *BinaryDilateImageFilter*() and *BinaryErodeImageFilter*() of the SimpleITK package [6]. After peritumoral zone extraction, all the zones were cropped to the brain outline using the MNI template, and manually in cases of originally cropped MRI images.

**Supplementary Fig. S1** Visualization of the chosen tumor-related zones. T= tumor zone (segmentation) (yellow), PTZ I = + 5 mm around tumor edge (blue), PTZ II = 0-10 mm outside tumor (red), PTZ III = 10-20 mm outside tumor (purple)

**S. 1.3. Methods: Included features from pyradiomics**

shape_Elongation

shape_Flatness

shape_LeastAxisLength

shape_MajorAxisLength

shape_Maximum2DDiameterColumn

shape_Maximum2DDiameterRow

shape_Maximum2DDiameterSlice

shape_Maximum3DDiameter

shape_MeshVolume

shape_MinorAxisLength

shape_Sphericity

shape_SurfaceArea

shape_SurfaceVolumeRatio

shape_VoxelVolume

firstorder_10Percentile

firstorder_90Percentile

firstorder_Energy

firstorder_Entropy

firstorder_InterquartileRange

firstorder_Kurtosis

firstorder_Maximum

firstorder_MeanAbsoluteDeviation

firstorder_Mean

firstorder_Median

firstorder_Minimum

firstorder_Range

firstorder_RobustMeanAbsoluteDeviation

firstorder_RootMeanSquared

firstorder_Skewness

firstorder_TotalEnergy

firstorder_Uniformity

firstorder_Variance

glcm_Autocorrelation

glcm_ClusterProminence

glcm_ClusterShade

glcm_ClusterTendency

glcm_Contrast

glcm_Correlation

glcm_DifferenceAverage

glcm_DifferenceEntropy

glcm_DifferenceVariance

glcm_Id

glcm_Idm

glcm_Idmn

glcm_Idn

glcm_Imc1

glcm_Imc2

glcm_InverseVariance

glcm_JointAverage

glcm_JointEnergy

glcm_JointEntropy

glcm_MCC

glcm_MaximumProbability

glcm_SumAverage

glcm_SumEntropy

glcm_SumSquares

gldm_DependenceEntropy

gldm_DependenceNonUniformity

gldm_DependenceNonUniformityNormalized

gldm_DependenceVariance

gldm_GrayLevelNonUniformity

gldm_GrayLevelVariance

gldm_HighGrayLevelEmphasis

gldm_LargeDependenceEmphasis

gldm_LargeDependenceHighGrayLevelEmphasis

gldm_LargeDependenceLowGrayLevelEmphasis

gldm_LowGrayLevelEmphasis

gldm_SmallDependenceEmphasis

gldm_SmallDependenceHighGrayLevelEmphasis

gldm_SmallDependenceLowGrayLevelEmphasis

glrlm_GrayLevelNonUniformity

glrlm_GrayLevelNonUniformityNormalized

glrlm_GrayLevelVariance

glrlm_HighGrayLevelRunEmphasis

glrlm_LongRunEmphasis

glrlm_LongRunHighGrayLevelEmphasis

glrlm_LongRunLowGrayLevelEmphasis

glrlm_LowGrayLevelRunEmphasis

glrlm_RunEntropy

glrlm_RunLengthNonUniformity

glrlm_RunLengthNonUniformityNormalized

glrlm_RunPercentage

glrlm_RunVariance

glrlm_ShortRunEmphasis

glrlm_ShortRunHighGrayLevelEmphasis

glrlm_ShortRunLowGrayLevelEmphasis

glszm_GrayLevelNonUniformity

glszm_GrayLevelNonUniformityNormalized

glszm_GrayLevelVariance

glszm_HighGrayLevelZoneEmphasis

glszm_LargeAreaEmphasis

glszm_LargeAreaHighGrayLevelEmphasis

glszm_LargeAreaLowGrayLevelEmphasis

glszm_LowGrayLevelZoneEmphasis

glszm_SizeZoneNonUniformity

glszm_SizeZoneNonUniformityNormalized

glszm_SmallAreaEmphasis

glszm_SmallAreaHighGrayLevelEmphasis

glszm_SmallAreaLowGrayLevelEmphasis

glszm_ZoneEntropy

glszm_ZonePercentage

glszm_ZoneVariance

ngtdm_Busyness

ngtdm_Coarseness

ngtdm_Complexity

ngtdm_Contrast

ngtdm_Strength

**S. 1.4. Methods: Radiomics feature selection and learning**

LASSO-Cox regression was implemented using the following functions: *CoxnetSurvivalAnalysis*(l1_ratio=1.0) and *GridSearchCV*() from sksurv [5] and sklearn [7] packages.

**S. 1.5. Methods: SHAP**

SHAP [8] was applied to visualize each feature’s value distribution within the test set of patients. SHAP also visualizes the importance of each feature in each survival model by the features’ contribution to the model’s output.

The following SHAP functions were utilized for interpretability: *Explainer*() and *plots.beeswarm*(). To add explanation of feature value colors in the plots, *TextArea*(), *HPacker*() and *AnchoredOffsetbox*() functions were used from the matplotlib.offsetbox package [2].

**S. 1.6. Methods: Survival model combination**

The combined models were built as the combination of optimal features within the selected radiomics model and features from each clinical model separately. The weights of features in the combined models were re-trained on the training set using Cox regression to obtain optimal weights for the new combination of features.

**S. 1.7. Methods: C-index distribution**

C-indexes were calculated using the *concordance_index_ipcw()* and *concordance_index_censored()* functions from the sksurv.metrics package [5].

The c-index distribution was built on 1000 sets of patient predictions, obtained by bootstrapping with replacement of the prediction set, separately for training and test set. The same bootstrapped IDs in the 1000 sets of predictions were used for each c-index variant to obtain comparable c-index value distributions. The following function was applied: *random.choice()* from the numpy package [3].

**S. 1.8. Methods: Risk threshold**

The risk threshold was selected by choosing the maximal test statistic value of log-rank test among possible risk thresholds on the entire cohort, while upholding a minimum group size of n=68 (33% of the cohort). The log-rank test was implemented using the *logrank_test*() function from the lifelines.statistics package [9].

Kaplan-Meier plots were done using the *KaplanMeierFitter*() from the lifelines package.

**S. 2.1. Results: Patient characteristics**

| **Variable** | **All patients**  **(n=207)** |
| --- | --- |
| Age at surgery, median (Q1, Q3) | 40.0 (32.0, 50.0) |
| Age > 40, n (%) | 99 (47.8) |
| KPS^a^ < 80 at admission, n (%) | 36 (17.4) |
| Tumor volume, ml, median (Q1, Q3) | 54.3 (24.3, 95.2) |
| Surgery |  |
| Biopsy, n (%) | 18 (8.7) |
| Partial resection, n (%) | 147 (71.0) |
| Complete resection, n (%) | 42 (20.3) |
| WHO classification |  |
| Grade 2, n (%) | 143 (69.1) |
| Grade 3, n (%) | 64 (30.9) |
| Astrocytoma, n (%) | 107 (51.7) |
| CDKN homozygote deletion |  |
| Retained, n (%) | 58 (28.0) |
| Not analysed/ Excluded or uncertain in methylation analysis, n (%) | 49 (23.7) |
| Oligodendroglioma, n (%) | 100 (48.3) |
| Oncological treatment |  |
| Radiotherapy (within 6 months), n (%) | 114 (55.1) |
| Chemotherapy (within 6 months), n (%) | 98 (47.3) |
| Survival |  |
| Deceased, n (%) | 51 (24.6) |
| Censored, n (%) | 156 (75.4) |

^a^Karnofsky performance status scale.

**Supplementary Table S1.** Demographics, patient characteristics, tumor characteristics, treatment and survival of the entire cohort.

**S. 2.2. Results: Harrell’s c-index**

| **Survival model** | **Selected features**  **N** | **Training**  **Harrell’s c-index,**  **median (IQR)** | **Test**  **Harrell’s c-index,**  **median (IQR)** |
| --- | --- | --- | --- |
| **Preoperative Clinical** | 1 | 0.706 (0.062) | 0.704 (0.088) |
| **Full Clinical** | 3 | 0.724 (0.065) | 0.723 (0.094) |
| **Radiomics** Tumor | 4 | 0.740 (0.052) | 0.760 (0.075) |
| **Radiomics** PTZ +5mm | 4 | 0.729 (0.057) | 0.677 (0.097) |
| **Radiomics** PTZ 0-10mm | 1 | 0.743 (0.054) | 0.740 (0.083) |
| **Radiomics** PTZ 10-20mm | 30 | 0.891 (0.042) | 0.707 (0.099) |
| **Combined**: Preop Clinical + Radiomics | 1 + 4 | 0.752 (0.049) | 0.780 (0.070) |
| **Combined**: Full Clinical + Radiomics | 3 + 4 | 0.770 (0.052) | 0.808 (0.069) |

**Supplementary Table S2.** Survival model results using Harell’s c-index performance evaluation for each model on the training set (n=144) and test set (n=63).

**S. 2.3. Results: Sensitivity analysis on full clinical model**

| **Survival model** | **Selected features**  **N** | **Training**  **Harrell’s c-index,**  **median (IQR)** | **Test**  **Harrell’s c-index,**  **median (IQR)** | **Test**  **Uno’s c-index**  **median (IQR)** | **Test**  **Uno’s at t < 5y**  **median (IQR)** |
| --- | --- | --- | --- | --- | --- |
| **Sensitivity analysis: Full clinical** (preop volume, *postop* vo*lume*, tumor subtype) | 3 | 0.746 (0.081) | 0.727 (0.116) | 0.631 (0.131) | 0.745 (0.151) |

**Supplementary Table S3.** Full clinical survival model results, using postoperative tumor volume instead of extent of resection. The postoperative tumor volume was available for a smaller subset of patients: training set (n=129) and test set (n=57).

**S. 2.4. Results: Optimal radiomics features on tumor area**

FLAIR_shape_Maximum2DDiameterSlice

FLAIR_shape_Maximum3DDiameter

FLAIR_glrlm_RunLengthNonUniformity

T1c_glrlm_RunLengthNonUniformity

**S. 2.5. Results: Additional value of radiomics diameter and heterogeneity features separately**

| **Survival model** | **Selected features**  **N** | **Training**  **Harrell’s c-index,**  **median (IQR)** | **Test**  **Harrell’s c-index,**  **median (IQR)** | **Test**  **Uno’s c-index**  **median (IQR)** | **Test**  **Uno’s at t < 5y**  **median (IQR)** |
| --- | --- | --- | --- | --- | --- |
| **Combined:** Full Clinical + Radiomics diameter features | 3 + 2 | 0.759 (0.054) | 0.818 (0.071) | 0.768 (0.115) | 0.825 (0.088) |
| **Combined**: Full Clinical + Radiomics heterogeneity features | 3 + 2 | 0.747 (0.062) | 0.733 (0.089) | 0.728 (0.142) | 0.722 (0.120) |

**Supplementary Table S4.** Model combination results by adding radiomics diameter and heterogeneity features separately to the full clinical model. Training set (n=144) and test set (n=63).

**S. 2.6. Results: Risk groups**

| **Variable** | **High-risk  patients (n=71)** | **Low-risk patients (n=136)** | **p-value^a^** |
| --- | --- | --- | --- |
| Age at surgery, median (Q1, Q3) | 41.0 (33.0, 50.0) | 39.0 (32.0, 49.8) | 0.49 |
| Age > 40, n (%) | 37 (52.1) | 62 (45.6) | 0.38 |
| KPS^b^ < 80 at admission, n (%) | 23 (32.4) | 13 (9.6) | <0.01 |
| Tumor volume, ml, median (Q1, Q3) | 111.8 (69.7, 166.6) | 32.4 (16.8, 57.6) | <0.01 |
| Surgery |  |  |  |
| Biopsy, n (%) | 10 (14.1) | 8 (5.9) | 0.07 |
| Partial resection, n (%) | 57 (80.3) | 90 (66.2) | 0.04 |
| Complete resection, n (%) | 4 (5.6) | 38 (27.9) | <0.01 |
| WHO classification |  |  |  |
| Grade 2, n (%) | 48 (67.6) | 95 (69.9) | 0.75 |
| Grade 3, n (%) | 23 (32.4) | 41 (30.1) | 0.75 |
| Astrocytoma, n (%) | 57 (80.3) | 50 (36.8) | < 0.01 |
| Oligodendroglioma, n (%) | 14 (19.7) | 86 (63.2) | <0.01 |
| Oncological treatment |  |  |  |
| Radiotherapy (within 6 months), n (%) | 47 (66.2) | 67 (49.3) | 0.03 |
| Chemotherapy (within 6 months), n (%) | 38 (53.5) | 60 (44.1) | 0.24 |
| Survival |  |  |  |
| Censored, n (%) | 36 (50.7) | 120 (88.2) | < 0.01 |

^a^ Mann-Whitney U test, Fisher’s exact test, ^b^ Karnofsky performance status scale.

**Supplementary Table S.5.** Demographics, patient characteristics, tumor characteristics, treatment and survival of the high- and low-risk group patients within the entire cohort.

**References**

1. McKinney, W., *pandas: a foundational Python library for data analysis and statistics.* Python for high performance and scientific computing, 2011. **14**(9): p. 1-9.

2. Barrett, P., et al. *matplotlib--A Portable Python Plotting Package*. in *Astronomical data analysis software and systems XIV*. 2005.

3. Harris, C.R., et al., *Array programming with NumPy.* Nature, 2020. **585**(7825): p. 357-362.

4. Virtanen, P., et al., *SciPy 1.0: fundamental algorithms for scientific computing in Python.* Nature methods, 2020. **17**(3): p. 261-272.

5. Pölsterl, S., *scikit-survival: A Library for Time-to-Event Analysis Built on Top of scikit-learn.* Journal of Machine Learning Research, 2020. **21**(212): p. 1-6.

6. Lowekamp, B.C., et al., *The design of SimpleITK.* Frontiers in neuroinformatics, 2013. **7**: p. 45.

7. Pedregosa, F., et al., *Scikit-learn: Machine learning in Python.* the Journal of machine Learning research, 2011. **12**: p. 2825-2830.

8. Lundberg, S.M. and S.-I. Lee, *A unified approach to interpreting model predictions.* Advances in neural information processing systems, 2017. **30**.

9. Davidson-Pilon, C., *lifelines: survival analysis in Python.* Journal of Open Source Software, 2019. **4**(40): p. 1317.
